# Switchable Asymmetric Water Transport in Dense Nanocomposite Membranes

**DOI:** 10.1021/acsapm.3c02801

**Published:** 2024-02-01

**Authors:** Luca Grillo, Christoph Weder

**Affiliations:** Adolphe Merkle Institute, University of Fribourg, Chemin des Verdiers 4, 1700 Fribourg, Switzerland

**Keywords:** asymmetric transport, nanocomposite membrane, water-induced plasticization, water permeability, bioinspired

## Abstract

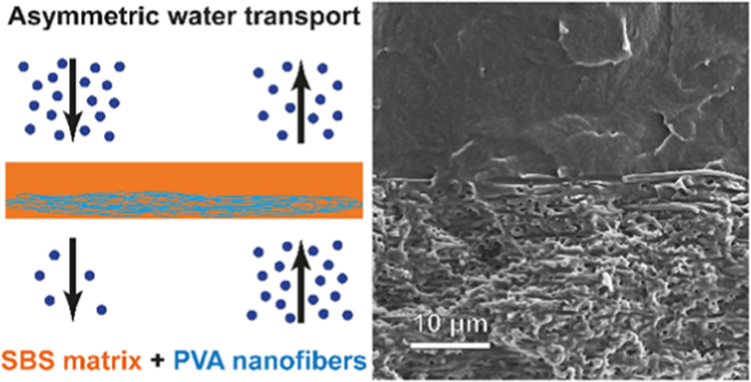

Directional water transport is technologically relevant in separation processes, functional clothing, and other applications. While asymmetric water transport characteristics are a vital feature of leaf cuticles, examples of artificial membranes that display this effect are limited. Here, we report compositionally asymmetric membranes that are based on hydrophobic poly(styrene)-*block*-poly(butadiene)-*block*-poly(styrene) (SBS) and hydrophilic poly(vinyl alcohol) (PVA) nanofibers and display directional water transport when a high relative humidity (RH) gradient is applied. This effect is caused by the asymmetric structure of the membrane and the fact that the water permeability of PVA depends on the water pressure applied and the extent of plasticization that it causes. The transport characteristics can be tuned by varying the composition of the membranes. Such materials with switchable asymmetric water transport may be useful for smart packaging applications in which the take-up or release of water is regulated as needed.

## Introduction

The first example of asymmetric mass transport dates back to 1941,^1^ when Hurst reported that the water evaporation rate through the cuticle of *Calliphora* larvae depends strongly on the transport direction. However, the fundamental principles that govern this phenomenon were formulated only a decade later by Rogers and co-workers, who established the conditions for directional mass transport in artificial multilayer polymeric membranes.^2^ After this pioneering study, several attempts have been made to model and predict the transport properties of asymmetric membranes, both with laminated and graded compositions.^3−7^ Nevertheless, asymmetric transport through dense heterogeneous membranes has not been widely explored.^8^ Studies on asymmetric water transport have mainly focused on directional wettability in fibrous and fabric systems for functional clothing applications,^9−11^ while the investigation of this feature in terms of directional moisture transport has received little attention,^12^ especially in dense membranes that could be useful for separation processes^13−15^ and packaging applications.^16^ The possibility of directionally regulating the water transport in smart packaging materials could be particularly useful for the storage of fruits and vegetables or other goods requiring good control of the hydration level to preserve their quality.^16^

According to membrane theory, directional mass transport occurs if two specific criteria are met. The first is that the membrane must have a spatially heterogeneous structure in terms of chemical composition or physical characteristics, i.e., the extent of crystallinity or the degree of cross-linking.^17^ The second is that at least one component must exhibit a permeability coefficient that depends on the vapor pressure of the permeant species.^2−8^ These two conditions have recently been demonstrated to promote asymmetric water transport in leaf cuticles, in which the compositionally graded structure and the water-induced plasticization of cutin, a polyester that is abundant in these membranes, result in preferential water transport from the outside to the inside.^18^ Inspired by this biological system, our group has recently developed compositionally graded membranes by combining a hydrophobic poly(styrene)-*block*-poly(butadiene)-*block*-poly(styrene) (SBS) matrix and hydrophilic cellulose nanocrystals (CNCs).^18^ A sedimentation process was employed to create a gradient in the CNC concentration along the transversal direction, resulting in asymmetric water transport.^18^ Similar materials, optionally with hydrophobized CNCs, were also used to prepare pervaporation membranes with directional transport characteristics.^19^

In the membranes mentioned above, the increased water permeability in the direction against the CNC concentration gradient was ascribed to the hydrophilic nature of the nanofiller.^18^ However, the high crystallinity of CNCs prevents water transport through these nanoparticles. Thus, transport occurs only along the hydrophilic CNC surface,^18,20^ and water sorption is restricted to the filler–polymer interface.^21−23^ To improve this aspect, we prepared and investigated similar membranes in which we replaced the CNCs with poly(vinyl alcohol) (PVA) nanofibers, whose amorphous domains also allow permeation *through* the nanofiller.^24−26^ Moreover, PVA, which in its dry state displays a high glass transition temperature of 80 °C, is known to undergo plasticization upon swelling and thus exhibits a water permeability that strongly depends on the water vapor pressure,^27−31^ which, as mentioned above, is a prerequisite to achieving asymmetric transport.^2−8^ Membranes having an asymmetric structure were produced by casting SBS solutions onto electrospun PVA mats and subsequently undergoing compression molding. The composition of the membranes was systematically varied to tune the magnitude of the water transport directionality. We demonstrate that the new materials display the water-switchable characteristics and the directional transport observed in biological membranes that inspired their design.

## Experimental Section

### Materials

Poly(styrene)-*block*-poly(butadiene)-*block*-poly(styrene) (SBS) (30 wt % styrene, *M*_w_ ∼ 140,000 by GPC) was purchased from Sigma-Aldrich (now Merck). Poly(vinyl alcohol) (PVA) (Mowiol 20–98, *M*_w_ ∼ 125,000), potassium chloride (KCl), and calcium chloride (CaCl_2_) (anhydrous, granular ∼1–2 mm) were supplied by Merck, and sodium chloride (NaCl) was purchased from Carl Roth. Tetrahydrofuran (THF) (99.8%, analytical reagent grade) and magnesium nitrate hexahydrate (Mg(NO_3_)_2_·6H_2_O) were purchased from Thermo Fisher Scientific and used without any further purification. In-house deionized (DI) water was used unless otherwise specified.

### Electrospinning of PVA

PVA was dissolved in DI water (10% w/w) by stirring at 90 °C for 3 h. The PVA solution was then transferred into a 20 mL glass syringe fitted with a stainless-steel needle of 21 gauge and subjected to electrospinning using a flow rate of 20 μL min^–1^ and an applied voltage of 15 kV. Aluminum foil was used as a stationary collector that was fixed at a distance of 15 cm away from the needle tip. The spinning time was set at 3.5 h for the fabrication of the PVA fiber mats.

### Fabrication of SBS-PVA_13_ Membranes

After electrospinning, the PVA mats were removed from the aluminum collector and cut into a circular shape to fit into poly(tetrafluoroethylene) (PTFE) Petri dishes with a diameter of 80 mm (the typical weight of the PVA mat was 220 mg). A concentrated solution of SBS in THF (15 wt %, 10 mL) was cast directly on the PVA mats. The films were left to dry overnight in a well-ventilated hood and subsequently dried in a vacuum oven at 85 °C for 24 h. The PVA content represents the weight fraction of PVA in the composite and was calculated from the weight of the PVA mat used and the weight of SBS in the casting solution. The membranes thus produced displayed heterogenous thicknesses and were further compression-molded in a Carver press between PTFE sheets with a pressure of 4 t for 7 min at 130 °C. Spacers of 250 μm were used to homogenize their thickness to a value of 248 ± 6 μm (see Table 1). The samples were then kept in a desiccator under vacuum before further characterization.

**Table 1:** Membrane Composition, Fabrication Method, and Thickness

sample code	fabrication method	film applier gap (mm)	total membrane thickness (μm)	thickness of the neat SBS layer^a^ (μm)
**SBS-PVA**_**13**_	solution casting	n.a.	248 ± 6	124 ± 19
**SBS-PVA**_**20**_	film applier	1.25	164 ± 4	87 ± 7
**SBS-PVA**_**23**_	film applier	0.75	108 ± 8	48 ± 8

a Estimated by analysis of the SEM images.

### Fabrication of SBS-PVA_20_ and SBS-PVA_23_ Membranes

After electrospinning, the PVA mats were removed from the aluminum collector and cut into rectangular shapes (7 × 10 cm) with a typical weight of 180 mg. The PVA mats were then transferred on a glass plate and a concentrated solution of SBS in THF (15 wt %, 6 mL) was poured on top of them and spread uniformly using a film applier (P.G.&T. Co., 8 Path Applicator). The composition of the nanocomposites was controlled by varying the gap clearance of the film applier during the solution casting step. More specifically, gaps of 1.25 mm and 0.75 mm were used to prepare SBS-PVA composites with 20 wt % (**SBS-PVA**_**20**_) and 23 wt % (**SBS-PVA**_**23**_) of PVA. The films were left to dry overnight in a well-ventilated hood, and after gently peeling them off the glass plates, they were further dried in a vacuum oven at 85 °C for 24 h. The PVA content represents the weight fraction of PVA in the composite and was calculated from the weight of the PVA mat used and the weight of SBS in the casting solution. The resulting membranes displayed a heterogenous thickness and were thus further compression-molded between Teflon sheets in a Carver press with a pressure of 4 t for 7 min at 130 °C. Spacers of 150 and 100 μm were used to control the thickness of **SBS-PVA**_**20**_ and **SBS-PVA**_**23**_, respectively, in order to obtain asymmetric composite membranes with the same ratio between the thicknesses of the neat SBS layer and the PVA-rich side of the film (see Table 1). The samples were then kept in a desiccator under vacuum before further characterization.

### Fabrication of Neat SBS and PVA Reference Membranes

The neat SBS reference membranes were prepared by compression molding of pellets in a Carver press. A specific amount of pellets was pressed between PTFE sheets with a pressure of 4 t for 7 min at 130 °C. For permeability tests and dynamic mechanical analysis (DMA), films with a thickness of 120–140 μm were prepared using 0.6 g of SBS pellets and spacers of 150 μm, while for tensile tests and water uptake measurements, we used 0.8 g of pellets and spacers of 250 μm to produce membranes with a thickness of ∼220 μm. Reference membranes of the neat PVA were prepared by solution-casting a solution of PVA in DI water (2.5 wt %, 40 mL) into a round PTFE Petri dish (diameter = 80 mm) and evaporating the solvent at 50 °C for 2 days. The resulting films, which were used for permeability tests, had a thickness of ∼200 μm.

### Thickness Measurements

All thickness measurements reported were carried out with a digital micrometer (IP 65, Mitutoyo).

### Scanning Electron Microscopy

The PVA mats were characterized as prepared. The neat SBS reference film and SBS-PVA composite membranes were cryo-fractured in liquid nitrogen to image their cross-sections. The samples were kept in a desiccator before imaging; after removal from the desiccator, they were sputtered with a 4 nm-thick gold layer (Cressington 208HR high-resolution sputter coater, U.K.). The coated samples were analyzed by scanning electron microscopy (SEM) using a Tescan Mira3 LM FE at 5 kV. The diameter of the PVA nanofibers was determined by analyzing at least four independent SEM images with image analysis software ImageJ, measuring the width of at least 20 individual nanofibers for each image. The thickness of the PVA-free SBS layer of the SBS-PVA composite membranes was also evaluated with ImageJ by analyzing at least eight SEM images for each composition and measuring the thickness at 10 different locations for each image.

### Thermogravimetric Analysis (TGA)

The thermal stability of the materials was investigated by thermogravimetric analysis using a Mettler-Toledo TGA/DSC 1 STARe system. Tests were performed under a nitrogen atmosphere in a temperature range of 25–550 °C with a heating rate of 10 °C min^–1^. TGA results were analyzed using STARe Evaluation software.

### Tensile Testing

Tensile tests were performed on a Zwick/Roell Z010 tensile tester equipped with a 200 N load cell. Dog bone-shaped samples were prepared from the membranes using a die cutter (Zwick/Roell, cutter length of 38.1 mm, path length of 22.25 mm, path width of 4.75 mm, width of 15.88 mm, and *R* = 3.2 mm). Stress–strain measurements were conducted under ambient conditions (23 °C, typical RH = 50%) using a strain rate of 50 mm min^–1^.

### Dynamic Mechanical Analysis (DMA)

DMA experiments were carried out on a TA Instruments, model Q800 dynamic mechanical analyzer in tensile mode with a strain amplitude of 15 μm and at a frequency of 1 Hz. The samples were equilibrated for 5 min at −100 °C with liquid N_2_ before a temperature sweep from −100 to 150 °C (heating rate of 5 °C min^–1^) was carried out. Specimens were prepared by cutting strips of rectangular shapes, typically 15 × 5.3 mm, and kept in a desiccator under vacuum overnight before measurements. The reported glass transition temperatures (*T*_g_) of the membranes were determined from the maxima of the acquired tan δ curves.

### Water Permeability Measurements

Standard test methods for water vapor transmission of materials according to ASTM E96 (dry cup and wet cup methods) were used to measure the water permeability of the SBS and PVA reference films and the SBS-PVA composite membranes.^32^ The membranes were cut into round shapes with a diameter of 6.5 cm, and 10 thickness measurements at random locations were taken per sample. The membranes were preconditioned under the test conditions for at least 2 days before they were mounted on water-impermeable aluminum cups (Thwing-Albert, U.S.A., 3/4 in. EZ Cup) having a test area (*A*) of 31.7 cm^2^ containing CaCl_2_ (dry cup method) or DI water (wet cup method). The cups were placed in a ventilated incubator set at 25 °C. For the dry cup method, a steady relative humidity at the donor side (RH_D_) was created by placing saturated salt solutions in the incubator. More specifically, solutions of Mg(NO_3_)_2_·6H_2_O (RH_D_ = ∼60%), NaCl (RH_D_ = ∼75%), or KCl (RH_D_ = ∼85%) were used.^33^ For the wet cup method, a large amount of CaCl_2_ (500 g) was placed in the incubator to keep the receiver compartment (RH_R_) at low relative humidity. The cups were removed in regular time intervals (1 < Δ*t* < 48 h) and weighed to evaluate the water vapor transmission rate (WVTR) from the slope of the change in mass of the assembly (*g*) as a function of time (*t*), divided by the transport area (*A*), as shown in eq 1
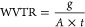
1

At least five data points over at least 24 h were analyzed for each linear regression. Eq 2 was used to calculate the water permeability (WP)
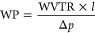
2where *l* and Δ*p* are the average membrane thickness and the water vapor pressure difference between the two sides of the membrane, respectively. The water permeability measurements were carried out in two directions, i.e., each side of the composite membranes facing the water donor compartment once. The reported values are the mean and standard deviation of experiments carried out on *n* = 4 membranes for each composition. The asymmetric transport was expressed in terms of the asymmetry factor (AF), which was measured using eq 3
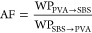
3where WP_PVA→SBS_ and WP_SBS→PVA_ correspond to the water permeability, WP, evaluated in the direction from the PVA-rich side to the neat SBS layer and vice versa, respectively.

### Water Uptake Measurements

Water uptake experiments were conducted at room temperature (23 °C). The neat SBS reference membranes and the SBS-PVA composite membranes were dried and cut into square samples of 1 × 1 cm^2^, and their initial dry weight (*M*_0_) was determined before immersing them in DI water (7 mL). The aqueous swelling was monitored gravimetrically by weighing the samples regularly until the equilibrium wet weight (*M*_∞_) was reached, typically after 96 h. The equilibrium water uptake was calculated with eq 4. The reported values are the mean and the standard deviation of *n* = 3 measurements for each composition.
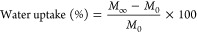
4

## Results and Discussion

### Fabrication and Characterization of SBS-PVA Composite Membranes

The first step in the fabrication process of the SBS-PVA composite membranes involves the preparation of PVA nanofibers by electrospinning. The fibrous PVA mats, which had a thickness of ca. 200 μm, were then either placed into a PTFE mold into which a solution of SBS in THF was cast over them or coated with the SBS solution using a film applier. By varying the process and the gap clearance of the film applier, the thickness of the SBS layer was changed to prepare SBS-PVA composites containing 13, 20, or 23 wt % PVA (sample code **SBS-PVA**_**xx**_, where xx is the weight fraction of PVA). After drying, the composite films were compression-molded at 130 °C to produce smooth membranes with a homogenous thickness that varied between 100 and 250 μm (Table 1), depending on the composition of the composite membranes. The process conditions were empirically varied to produce membranes in which the thickness of the PVA-free, neat SBS layer (estimated by SEM image analysis; see below) corresponds to ca. 50% of the total membrane thickness. This process allows a much better control over the asymmetric distribution of the hydrophilic component in the hydrophobic matrix in comparison to previously investigated CNC-based nanocomposites, which were prepared by a particular sedimentation process that afforded a gradient in the CNC concentration along the transversal direction of the membranes.^18^ In principle, the fabrication method developed in this work can be generalized and exploited with other combinations of nanofibers and polymer solutions to generate various types of asymmetric nanocomposite membranes.^34,35^ PVA and SBS are used in pervaporation membranes,^15,19^ and we speculate that in graded structures such as the ones studied here, the SBS could serve as a protective layer and improve the separation performance of the hydrophilic PVA.^36^

**Figure 1:**
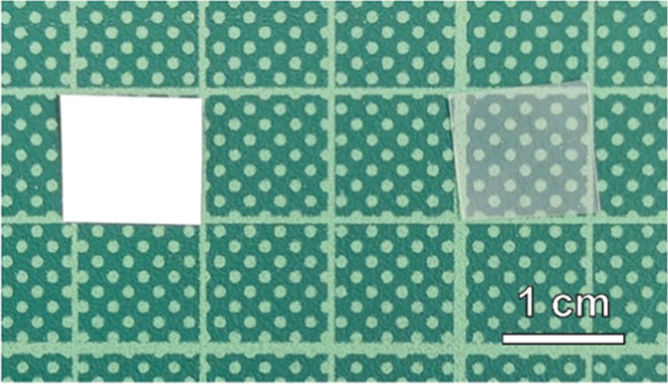
Pictures of an electrospun PVA mat (left) and a **SBS-PVA**_**13**_ composite film (right).

Reference membranes consisting of the neat SBS and the neat PVA, respectively, were produced by compression-molding SBS pellets and solvent-casting PVA. We also produced solvent-cast SBS (Figure S1a) and annealed PVA (Figure S1b) reference films to demonstrate that the processing history has no influence on the water transport characteristics of the neat reference membranes. Pictures of an electrospun PVA mat and a SBS-PVA composite film containing 13 wt % PVA (**SBS-PVA**_**13**_) are shown in Figure 1. While the porous PVA mat appears as a white film, on account of light scattering, the SBS-PVA composite is transparent and colorless, which indicates that the SBS completely fills the pores of the PVA scaffold.

**Figure 2:**
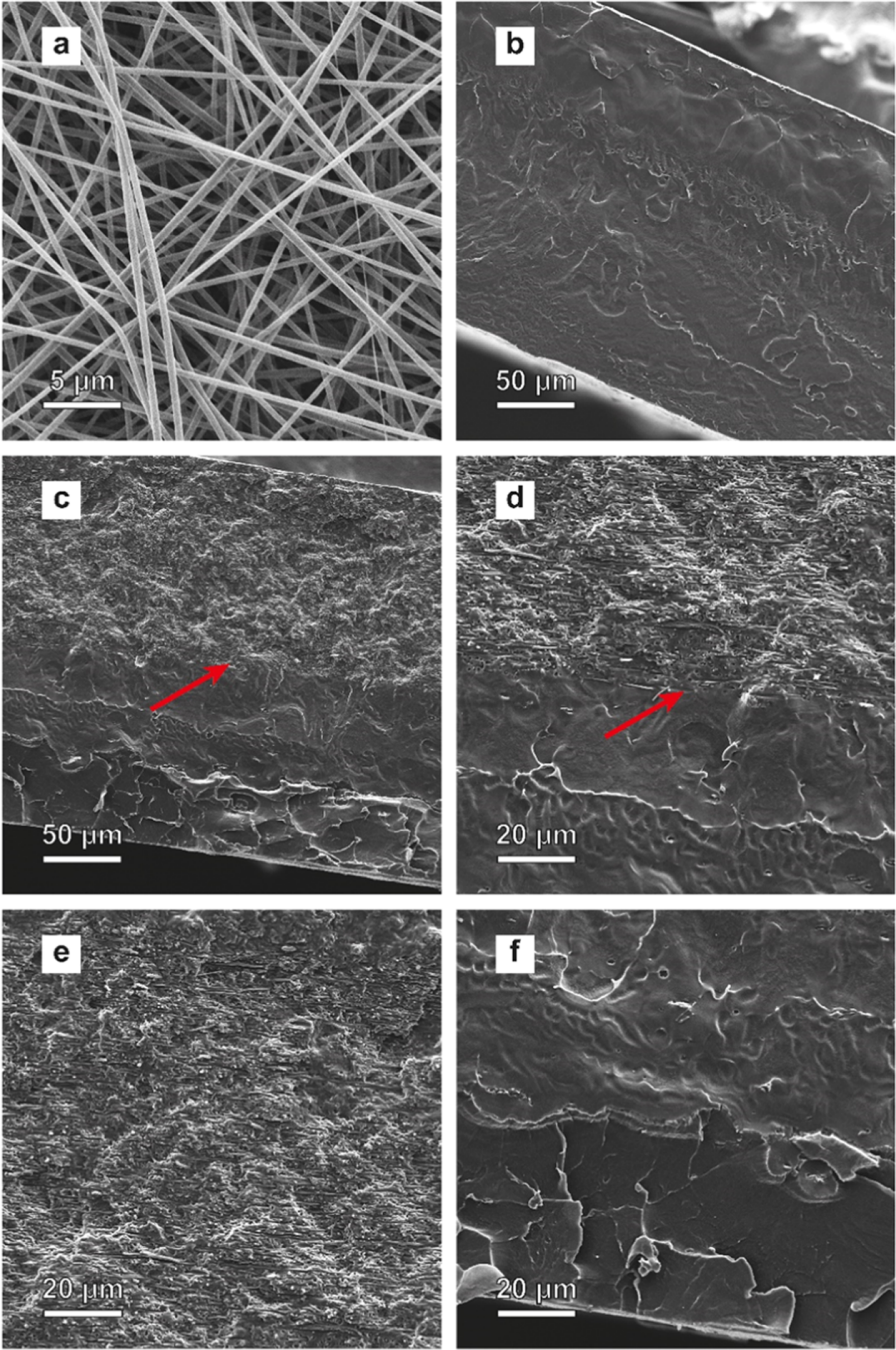
SEM images of (a) electrospun PVA mat, (b) cross-section of a neat SBS reference film, (c, d) cross-sections of a **SBS-PVA**_**13**_ composite membrane (red arrows indicate the interface between the PVA-rich side and the neat SBS layer), and magnifications of (e) PVA-rich side and (f) neat SBS layer of a **SBS-PVA**_**13**_ composite membrane.

The morphology of the neat PVA mats, a neat SBS reference film, and the SBS-PVA composite membranes was investigated by scanning electron microscopy (SEM) (Figures 2, S2–S3). The SEM image of the PVA mat shows a highly porous network of mostly straight PVA fibers that have an average width of 357 ± 59 nm (Figure 2a). While the cross-section of a cryo-fractured SBS reference film reveals a smooth, uniform morphology (Figure 2b), two different textures are observed in the corresponding images of the SBS-PVA membranes (Figures 2c–f, S2). The SEM images of the cross-sections of the composite membranes show that the PVA fibers are fully incorporated into the SBS matrix without any interfacial gaps between the two components (Figures 2c,d, S2a,b). The SEM images of the fractured cross-section further show that the PVA-rich halves of the membranes all have a rough appearance that is the result of the fracture of a heterogenous composite (Figures 2e, S2c), whereas the PVA-free areas have a smooth texture as the one shown in the reference film of neat SBS (Figures 2f, and S2d). The asymmetric morphology is further reflected by SEM images of the two different surfaces of the membranes (Figure S3), which show the absence (Figure S3a,c) or presence (Figure S2b,d) of embedded fibers. The thickness of the neat SBS layer of the composite membranes was determined by analyzing the SEM images using ImageJ (Table 1). As designed, all membranes are composed of equal portions that contain or are free of PVA nanofibers.

The thermal stability of the SBS-PVA composites and the individual components was determined by thermogravimetric analysis (TGA) (Figure S4). The neat SBS reference film shows the highest stability, with a degradation onset at around 420 °C, while the electrospun PVA mat starts to degrade at 340 °C. The PVA mat also shows a slight weight loss of 2% below 100 °C, which is related to the release of the absorbed moisture,^37^ even though the samples were thoroughly dried before the experiment. The TGA traces of the composite membranes show a two-step degradation process that recalls features of the degradation profiles of the individual components: a first weight loss step that is associated with the PVA nanofibers and increases in magnitude with the PVA content and a second step that is related to the degradation of the SBS matrix. Thus, the composite membranes are thermally stable at the processing temperature (130 °C).

### Mechanical Properties of SBS-PVA Composite Membranes

**Figure 3:**
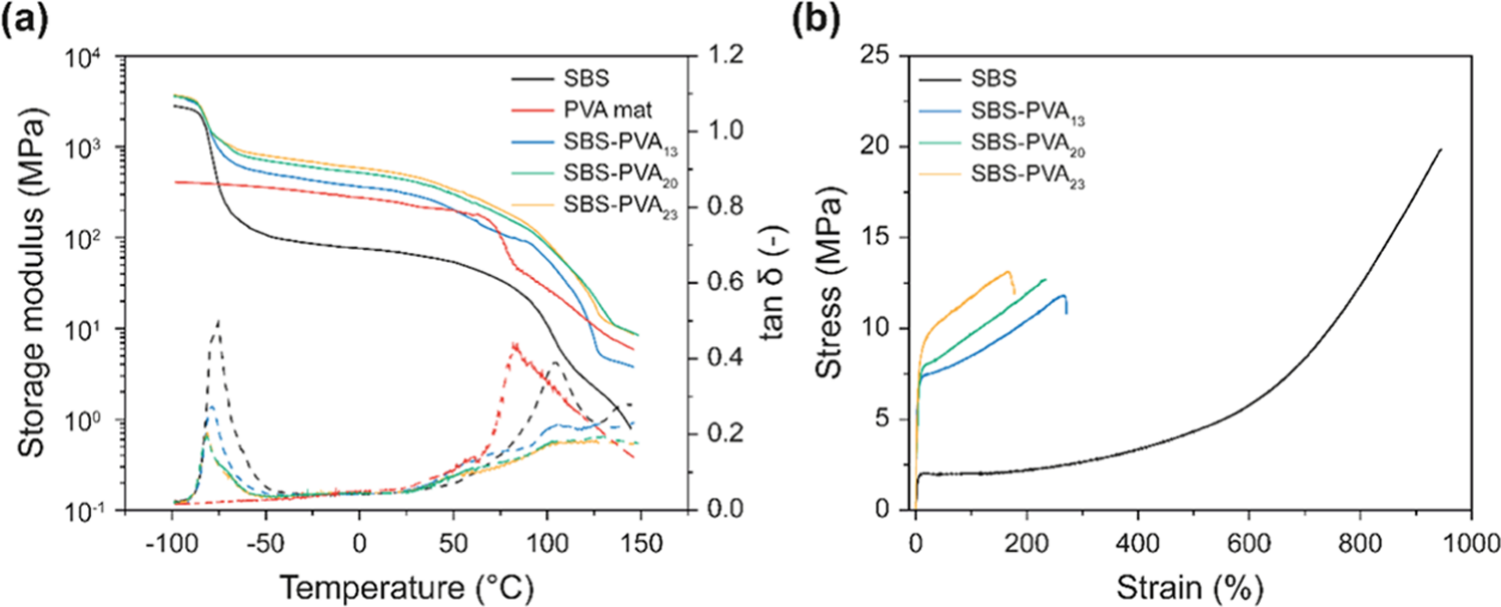
Representative (a) dynamic mechanical analysis (DMA) traces and (b) stress–strain curves (recorded at 23 °C) of the neat SBS and SBS-PVA nanocomposite membranes.

To probe how the incorporation of PVA nanofibers affects the mechanical properties of the composite materials, dynamic mechanical analyses (DMA) and tensile tests were carried out (Figure 3). The DMA tan δ-trace of a neat SBS reference film shows two well-defined peaks at ca. −76 and 105 °C, which correspond to the glass transition temperatures (*T*_g_) of domains formed by the poly(butadiene) (PB) and poly(styrene) (PS) blocks, respectively (Figure 3a, Table 2). Accordingly, the storage modulus (*E*′) trace shows a glassy regime where *E*′ is ca. 2.5 GPa, a rubbery regime between ca. −50 and 100 °C with a room-temperature *E*′ of 69 MPa (20 °C) and a dropping modulus above ca. 50 °C, before samples fail at ca. 150 °C. The DMA traces of the PVA mat reveal a glassy regime below the *T*_g_, in which the maximum of the tan δ-trace appears at ca. 84 °C. The fact that *E*′ (210 MPa at 20 °C) is much lower than expected for a glassy polymer is related to the porous nature of the PVA mat. Nevertheless, the DMA traces show that the PVA mat is stiffer than the SBS in its rubbery state. As a result, a significant reinforcement can be observed for the composites at different contents of PVA: the storage modulus at 20 °C is increased from 69 ± 14 (neat SBS) to 296 ± 28 (13 wt % of PVA) to 489 ± 37 (20 wt % of PVA) and 465 ± 38 (23 wt % of PVA). These values exceed the storage modulus of the PVA mat because the composite is no longer porous. The incorporation of the PVA nanofibers also results in a significant reduction of the peaks seen in the tan δ-traces, as a consequence of the restrained chain mobility of the SBS matrix in the presence of a rigid nanofiller.^15,38,39^

**Table 2:** Mechanical Properties of Neat SBS and SBS-PVA Nanocomposite Membranes

	*T*_g_^a^ [°C]					
sample code	PB	PS	storage modulus *E*′^a^ [MPa] (20 °C)	Young’s modulus^b^ [MPa]	elongation at break^b^ [%]	maximum stress^b^ [MPa]
**SBS**	–76	105	69 ± 14	54 ± 5	960 ± 15	20 ± 4
**SBS-PVA**_**13**_	–79	109	296 ± 28	386 ± 12	262 ± 14	12 ± 1
**SBS-PVA**_**20**_	–81	124	489 ± 37	425 ± 44	222 ± 35	12 ± 2
**SBS-PVA**_**23**_	–81	119	465 ± 38	423 ± 38	189 ± 15	12 ± 1

a Determined by dynamic mechanical analysis. The reported glass transition temperatures were determined from the maxima of the tan δ-curves.

b Determined by tensile tests at 23 °C. All data represent averages of *n* = 3 individual measurements ± standard deviation.

The reinforcing effect of the PVA fibers is also reflected in the tensile tests (Figure 3b). The stress–strain curves of the neat SBS reference film show a yield point at 5% strain and 1.7 MPa stress, extensive strain hardening, and high extensibility, with a failure strain of >900% (Table 2). The analysis of the low-strain regime results in Young’s modulus of 54 ± 5 MPa, which is of the same magnitude as *E*′. The introduction of the PVA nanofibers alters the mechanical behavior considerably; Young’s modulus is increased to ca. 400 MPa, the elongation at break is reduced to ca. 200%, and the failure stress drops from 20 to 12 MPa (Table 2). Nevertheless, the composites retain an appreciable extensibility, which we relate to the flexibility of the PVA mats.

### Characterization of Water Transport Characteristics

**Figure 4:**
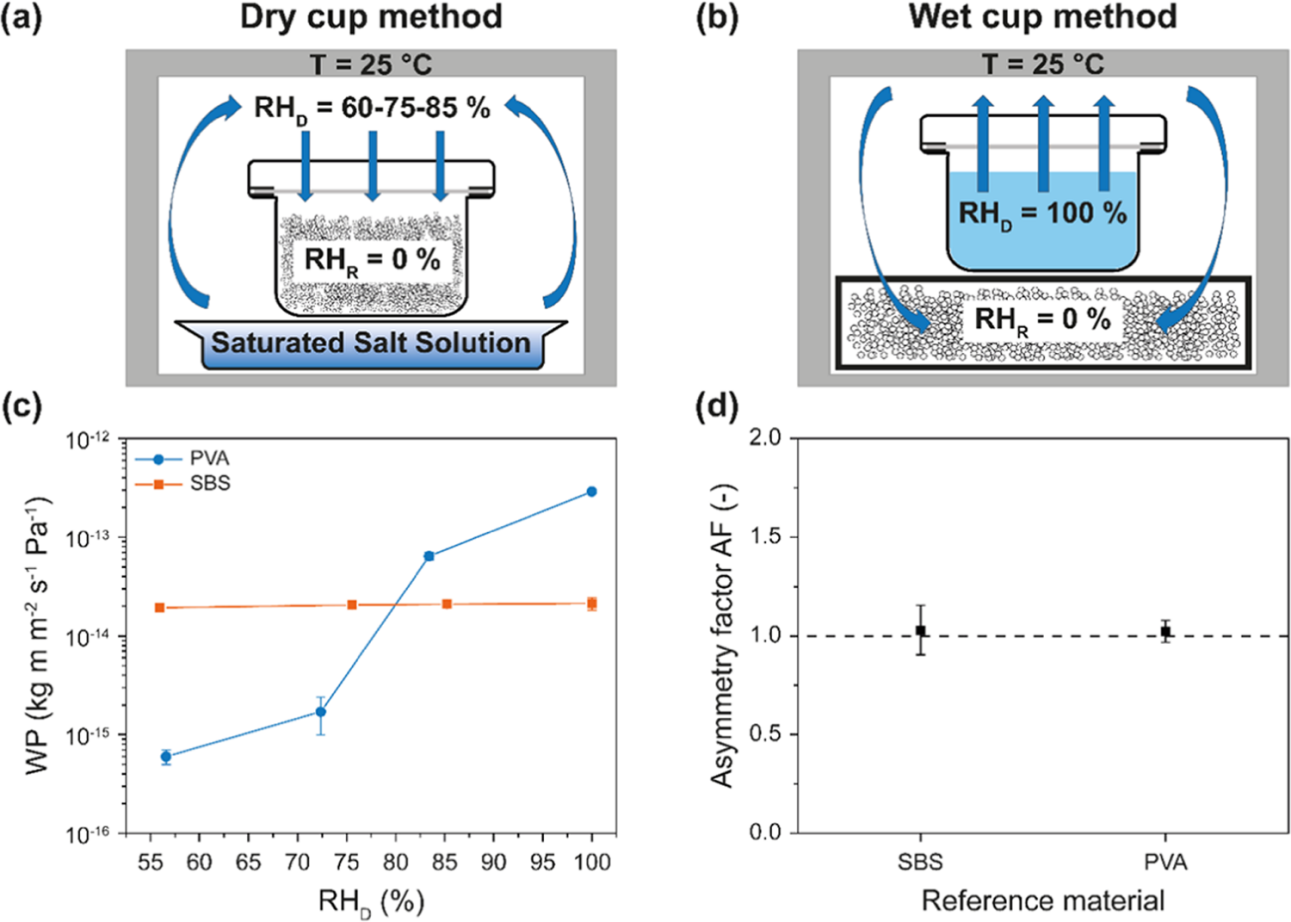
Schematic representation (not to scale) of (a) dry cup and (b) wet cup methods that were used to determine the water permeability (WP) of the various membranes at 25 °C. (c) WP of neat SBS and neat PVA reference membranes as a function of the relative humidity in the donor compartment (RH_D_). The wet cup method (b) was used for tests at RH_D_ = 100% with the dry cup method (a) for all other experiments. (d) Asymmetry factor (AF) in the reference films of neat SBS and neat PVA. Data shown in (d) were measured with the wet cup method (RH_D_ = 100%). Values are the mean ± s.d. of measurements carried out on *n* = 4 different membranes.

The water transport properties of the SBS-PVA membranes as well as those of neat SBS and PVA reference membranes were investigated as a function of the direction and relative humidity at the donor side (RH_D_) using gravimetric dry cup (for RH_D_ = 60, 75, and 85%) and wet cup (for RH_D_ = 100%) methods (Figure 4a,b). The water permeability (WP) of SBS reference membranes is practically independent of the relative humidity in the donor compartment, remaining almost constant at a value of ca. 2.05 × 10^–14^ kg m m^–2^ s^–1^ Pa^–1^ over the range of RH_D_ investigated, i.e., from 60 to 100% (Figure 4c). This is consistent with the fact that neither the polystyrene nor polybutadiene domains considerably sorb water, so that the transport properties remain essentially unchanged. By contrast, the WP of PVA references is strongly dependent on the conditions and increases by nearly 3 orders of magnitude from 5.99 ± 1.00 × 10^–16^ kg m m^–2^ s^–1^ Pa^–1^ at RH_D_ = 60% to 2.90 ± 0.08 × 10^–13^ kg m m^–2^ s^–1^ Pa^–1^ (RH_D_ = 100%) (Figure 4c). The strong dependence of the WP of PVA on the water vapor pressure is well known^27−31^ and is attributed to the plasticization effect that water has on this polymer. Plasticization increases the polymer chain mobility and free volume and thereby the permeant’s diffusion rate.^30,31^ Data recorded at RH_D_ = 100% further show that the water transport through the neat SBS and PVA membranes is in both cases symmetric, i.e., WP is the same, no matter which side of the membranes faces the donor compartment. Consequently, the asymmetry factor (AF), defined as the ratio of the water permeabilities measured in two opposite directions, is unity (Figure 4d).

**Figure 5:**
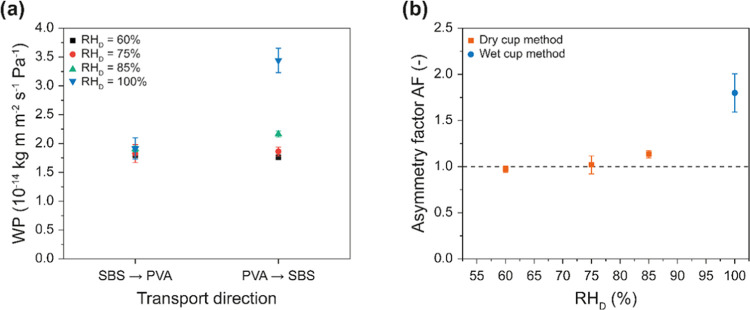
(a) Water permeability (WP) of **SBS-PVA**_**13**_ composite membranes at different RH_D_ values as a function of the transport direction. (b) Asymmetry factor (AF) of **SBS-PVA**_**13**_ composite membranes as a function of RH_D_, calculated from the data shown in (a). Reported values are the mean ± s.d. of measurements carried out on *n* = 4 different membranes.

Moving to the composite membranes, initial experiments were carried out on membranes with a PVA content of 13 wt %, a total thickness of 248 ± 6 μm, and a 124 ± 19 μm-thick neat SBS layer (**SBS-PVA**_**13**_). The WP measured in the direction from the SBS side to the PVA-rich side of the composite film (SBS → PVA) is relatively independent of RH_D_, assuming values of 1.81 ± 0.06–1.91 ± 0.19 × 10^–14^ kg m m^–2^ s^–1^ Pa^–1^ (Figure 5a). By contrast, when the transport direction is inverted and the PVA-rich side faces the donor (PVA → SBS), the WP increases with RH_D_ from 1.76 ± 0.03 to 3.44 ± 0.21 × 10^–14^ kg m m^–2^ s^–1^ Pa^–1^ (RH_D_ = 60 and 100%, respectively). A comparison of the AF values determined from these data (Figure 5b) shows that the water transport is symmetric at RH_D_ = 60 and 75% (AF = 0.97 ± 0.04 and 1.02 ± 0.10, respectively) but becomes asymmetric at higher RH_D_, reaching a maximum value of 1.80 ± 0.21 at RH_D_ = 100%. The data reflect that the transport remains symmetric as long as the PVA is not plasticized, i.e., at low RH or when the SBS side faces the donor. By contrast, when the PVA-rich side is exposed to a high humidity level, the water sorption is substantially increased (*vide infra*), and the originally glassy PVA is plasticized. The same principle to achieve asymmetric transport upon plasticization of a portion of a compositionally graded membrane is also at play in the biological membranes that inspired this system.^18^ The glassy-to-rubbery transition of PVA upon swelling allowed us to implement this switchable feature in the membranes presented here, which was not the case in the previously studied membranes containing water-transporting CNCs, which showed asymmetric water transport even at low RH, i.e., 75%, and without a strong dependence of the asymmetry factor with respect to the relative humidity.^18^

**Figure 6:**
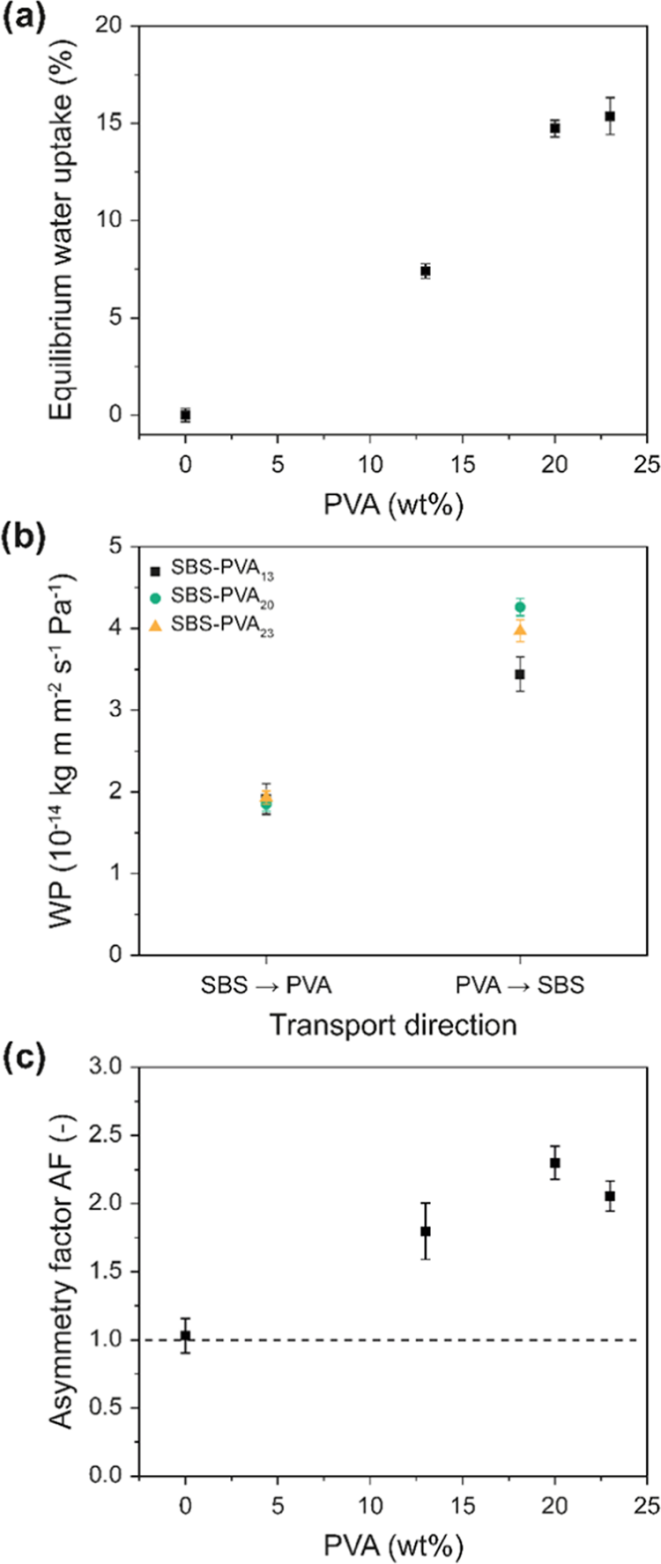
(a) Equilibrium water uptake of the composite membranes as a function of PVA content, expressed as mean ± s.d. of *n* = 3 samples. (b) WP of the composite membranes as a function of transport direction. (c) AF as a function of the PVA content. Data shown in (b, c) were measured with the wet cup method (RH_D_ = 100%) and are the mean ± s.d. on *n* = 4 membranes.

Considering that in the case of **SBS-PVA**_**13**_, the AF is most prominent at high RH_D_ (Figure 5b), the effect of the PVA content on the water transport characteristics was investigated using the wet cup method with RH_D_ = 100% (Figure 6). We first probed the equilibrium swelling of the various membranes upon immersion in water (Figure 6a). While the water uptake of the neat SBS is negligible (0.0 ± 0.3%), the extent of swelling increases with the PVA content and ranges from 7.4 ± 0.4 (**SBS-PVA**_**13**_) to 15.4 ± 0.9% (**SBS-PVA**_**23**_). Considering the hydrophobic nature of the SBS matrix, the water uptake is confined to the PVA nanofibers. Accordingly, as shown in Figure 6b, the water permeability is the same for all three compositions if the SBS side faces the donor compartment. Because the hydrophobic SBS-rich layer is an effective water barrier, the PVA phase is not sufficiently plasticized under this condition, and all three compositions display a similar WP of ca. 1.90 × 10^–14^ kg m m^–2^ s^–1^ Pa^–1^. By contrast, when the PVA-rich side faces the high-RH donor, the membranes swell in the PVA-rich compartment, and the WP increases from 3.44 ± 0.21 (**SBS-PVA**_**13**_) to 4.26 ± 0.11 × 10^–14^ kg m m^–2^ s^–1^ Pa^–1^ (**SBS-PVA**_**20**_). This is also reflected in an increase in the AF from 1.80 ± 0.21 to 2.30 ± 0.12 (Figure 6c). No further growth in the AF was observed when the PVA content was increased to 23 wt % (**SBS-PVA**_**23**_). This result seems to suggest that in compositions with a higher PVA content, the transport characteristics of the SBS layer may limit the AF.

### Analysis of the Data on the Basis of a Series Resistance Model

To study the impact of the different layers on the water transport properties of the SBS-PVA composites, we analyzed the data on the basis of a series resistance model. For this, we considered the membrane as a pseudobilayer structure consisting of a neat SBS layer and a second composite layer rich in PVA. By defining the resistance to water transport (*R*) through a membrane with eq 5, where *l* and *A* are the average thickness and the transport area of the membrane, respectively, the use of this simple model, also known as ideal laminate theory,^8^ allows the prediction of the total resistance of layered membranes from the sum of the resistances in the individual layers, which are considered as connected in series.^2,8,40^
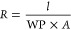
5

Considering the present membranes, the total resistance (*R*_M_) is given by the contributions of the SBS layer (*R*_SBS_) and of the PVA-rich nanocomposite (*R*_PVA-rich_), as shown in eq 6
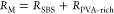
6

We exemplarily applied this model to the **SBS-PVA**_**23**_ composite membranes. We used the WPs determined for the composite membrane (Figure 6b) and the neat SBS (Figure 4c) and the thickness of the SBS layer estimated by the analysis of the SEM images (Table 1) to determine the overall resistance of the membrane *R*_M_ and the contribution of the SBS layer (*R*_SBS_) according to eq 5. Employing eq 6, we then derived the contribution of the PVA-rich portion of the membrane (*R*_PVA-rich_). The results are reported in Table 3, and the calculations are provided in Supporting Note 1.

**Table 3:** Water Permeability and Water Resistance Contributions Assessed in the **SBS-PVA**_**23**_ Composite Membranes

transport direction	water permeability^a^ [10^–14^ kg m m^–2^ s^–1^ Pa^–1^]	*R*_M_^a^ [MPa g^–1^ s^–1^]	*R*_SBS_^a^ [MPa g^–1^ s^–1^]	*R*_PVA-rich_^a^ [MPa g^–1^ s^–1^]
SBS → PVA	1.93 ± 0.08	1764 ± 213	687 ± 122	1077 ± 202
PVA → SBS	3.97 ± 0.13	856 ± 80	687 ± 122	169 ± 88

a Measured with the wet cup method (RH_D_ = 100%). Reported values are the mean ± s.d. of calculations carried out on *n* = 4 different membranes.

Table 3 shows that (for RH_D_ = 100%) the orientation-dependent *R*_M_ is much higher when measured in the direction from the hydrophobic SBS side to the PVA-rich side than in the opposite direction. Since the WP of SBS does not depend on the water vapor pressure (Figure 4c), the resistance of the SBS layer does not depend on the transport direction and is mainly governed by the thickness of the layer itself. By contrast, the resistance of the PVA-rich portion strongly depends on the transport direction. When the SBS side faces the donor compartment (RH_D_ = 100%), the hydrophobic SBS layer presents a barrier that prevents the swelling of the PVA, which in the dry state shows a very low WP, i.e., 5.99 ± 1.00 × 10^–16^ kg m m^–2^ s^–1^ Pa^–1^ at RH_D_ = 60% (Figure 4c), in the PVA-rich portion. Under these conditions, the latter shows a higher resistance to water transport than that of neat SBS (Table 3). When the transport direction is inverted and the PVA-rich side is exposed to water, the PVA is plasticized and its water permeability increases up to 2.90 ± 0.08 × 10^–13^ kg m m^–2^ s^–1^ Pa^–1^ (RH_D_ = 100%). Consequently, the resistance in the PVA-rich portion drastically decreases (Table 3). Under wet conditions, the transport of water in the PVA-rich side is facilitated by the plasticization of the PVA fibers, while the main resistance is given by the SBS layer. Thus, this series resistance model makes it clear that the asymmetric transport characteristics of the composite membranes are governed by the different resistances encountered in the PVA-rich side. The modeling also shows that the magnitude of this directionality could be improved by increasing the contrast in the resistance of the PVA-rich side *R*_PVA-rich_ upon swelling and by reducing *R*_SBS_, using, for example, a thinner SBS layer. The SBS layer should be thick enough to prevent swelling of the PVA-rich side but not too thick to cause a high resistance to water transport in the direction PVA → SBS.

In an effort to better understand the different resistances that the PVA-rich composite layer exhibits in the dry and wet states, we calculated the water permeability of this layer (WP_PVA-rich_) from the water permeability of the neat PVA (WP_PVA_) and the neat SBS (WP_SBS_) on the basis of eq 7(3) and the volume fraction of PVA in the PVA-rich side of the membrane (*v*_PVA_).
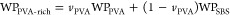
7

**Figure 7:**
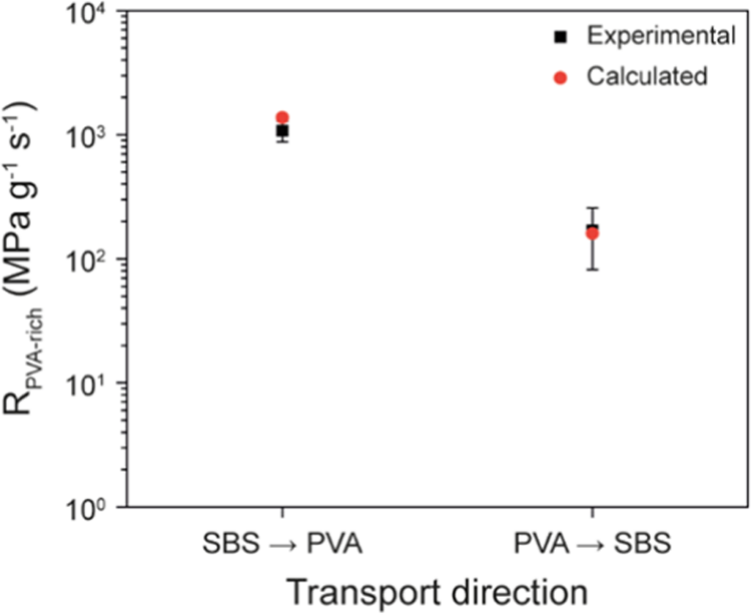
Resistance to water transport in the PVA-rich side (*R*_PVA-rich_) of the **SBS-PVA**_**23**_ nanocomposite membranes. Shown are the experimental values presented in Table 3 and the calculated values shown in Table 4. The experimental values are reported as the mean ± s.d. of *n* = 4 different membranes.

Considering the weight fraction of PVA in the total membrane and assuming that the PVA nanofibers are present only in the PVA-rich side, we estimated a value of 0.36 for *v*_PVA_ in the nanocomposite layer of **SBS-PVA**_**23**_ (see calculations in Supporting Note 2). While WP_SBS_ is independent of the water vapor pressure, WP_PVA_ changes from a dry value to a wet value (Figure 4c). Taking this variation of WP_PVA_ into consideration, we calculated the water permeability of the PVA-rich side with the PVA being either in a dry or water-swollen state. Considering the overall geometry of the membranes, these two conditions of the PVA-rich side correspond to the transport direction SBS → PVA, when the swelling of PVA cannot occur, and PVA → SBS, when the PVA plasticizes. Using the definition of the resistance given in eq 5, we then evaluated the corresponding resistance of the PVA-rich side *R*_PVA-rich_ using the thickness established by the analysis of the SEM images (Table 1). The results are shown in Table 4, and the calculations are provided in Supporting Note 2. Gratifyingly, the experimentally determined and calculated values match very nicely, as shown in Figure 7. Thus, the water permeability of the PVA-rich side of the present membranes, in both the dry and wet states, is well described by eq 7. On this basis, one can predict that the asymmetry factor of the present membranes could be further maximized by increasing the volume fraction of PVA in the PVA-rich side of the membrane.

**Table 4:** Theoretical Values of the Water Permeability (WP_PVA-rich_) and Resistance (*R*_PVA-rich_) of the PVA-Rich Layer of the **SBS-PVA**_**23**_ Composite Membrane in Different Transport Directions (RH_D_ = 100%)

transport direction	WP_PVA-rich_ [10^–14^ kg m m^–2^ s^–1^ Pa^–1^]	*R*_PVA-rich_ [MPa g^–1^ s^–1^]
SBS → PVA	1.38	1374
PVA → SBS	11.85	160

## Conclusions

In summary, we have shown that the infiltration of SBS into a porous mat of PVA nanofibers through solution casting allows the fabrication of dense and transparent membranes, where the fibers are preferentially located on one side of the nanocomposites. The incorporation of this continuous nanofiller impacts the mechanical properties of the nanocomposites with respect to the neat SBS, as reflected by the increase of Young’s modulus and the considerable changes in tensile behavior. Nevertheless, the composites retain an appreciable elongation at break, which we relate to the flexibility of the PVA mats. The selective swelling and consequent plasticization of the PVA fibers embedded in the asymmetric structure induced the directional transport of water upon switching. Under dry conditions, i.e., below ca. 75% RH, the water permeation is symmetric. However, when the RH on the PVA-rich side of the membranes exceeds 75%, the membranes swell and the permeability of PVA exceeds that of the matrix. This effect renders the transport of water asymmetric with a preferential direction following the hydrophilicity gradient within the nanocomposites. The maximum AF of 2.3 is comparable to the value reported recently for asymmetric membranes of the same SBS matrix and cellulose nanocrystals (2.5)^18^ and appears to be limited by the moderate change in the water resistance of the PVA-rich side of the membranes upon swelling. Based on the series resistance model and the prediction of the resistance in the PVA-rich side adopted in this study, we expect that higher AF values can be obtained by a bilayer structure consisting of PVA that is coated with a thin layer of SBS. However, while such a bilayer system should provide an increased directionality in water transport, the incompatibility between hydrophobic SBS and hydrophilic PVA is likely to cause delamination problems, which were avoided in the nanocomposite membranes presented here. We thus speculate that compositionally graded composite membranes that feature a very thin SBS layer and a PVA-rich composite layer represent an interesting target.

## Data Availability

The dataset underlying the findings of this article can be found at https://doi.org/10.5281/zenodo.10479231.

## Supporting Information Available

Water permeability of the reference films as a function of the fabrication steps, additional SEM images, thermogravimetric analysis, calculations of the resistance to water transport in the **SBS-PVA**_**23**_ composite membranes, and theoretical evaluation of the contribution of the PVA-rich side to the resistance of the **SBS-PVA**_**23**_ composite membranes (PDF)
