# Supplementary material for: Switchable Asymmetric Water Transport in Dense Nanocomposite Membranes
Source: ACS Appl Polym Mater. 2024 Feb 1;6(4):2243–52. doi: 10.1021/acsapm.3c02801 (PMC10897881; doi:10.1021/acsapm.3c02801)
Supplement: Supplementary file 1 — ap3c02801_si_001.pdf [file ap3c02801_si_001.pdf]

Supporting Information

Switchable asymmetric water transport in dense nanocomposite membranes

*Luca Grillo, Christoph Weder**

*christoph.weder@unifr.ch

Adolphe Merkle Institute, University of Fribourg, Chemin des Verdiers 4, 1700 Fribourg, Switzerland

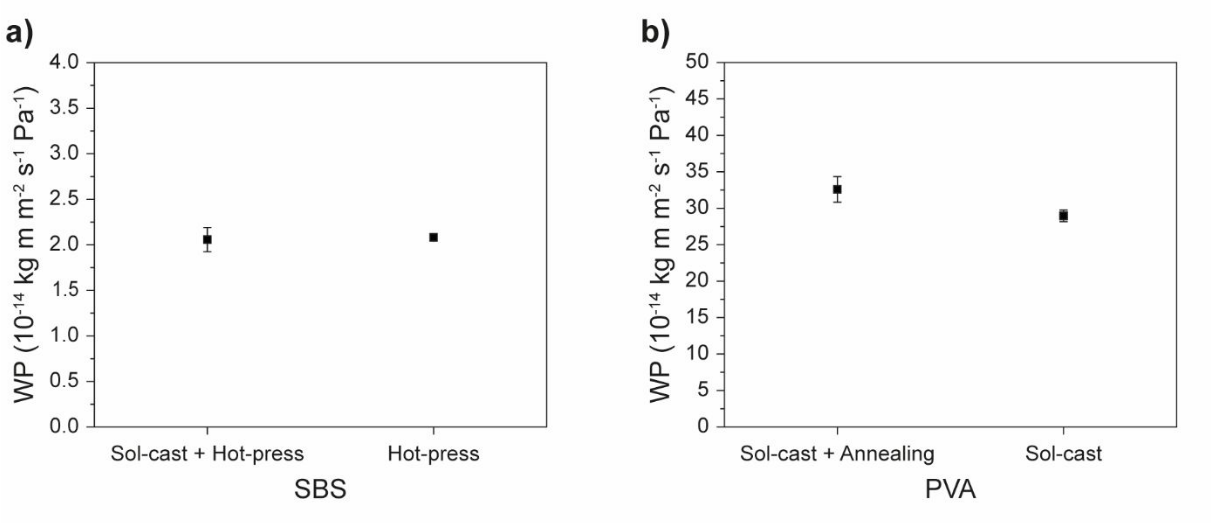

Figure S1: (a) Effect of the processing conditions on the WP of reference films of neat SBS; data were collected with the dry cup method (RH_D = 75%) and acquired on films that were either solution cast and hot-pressed or only hot-pressed. (b) Effect of the processing conditions on the WP of reference films of neat PVA; data were collected with the wet cup method (RH_D = 100%) and acquired on films that were either solution cast and annealed or only solution-cast. All reported values are the mean ± s.d. of measurements on n = 4 different membranes.

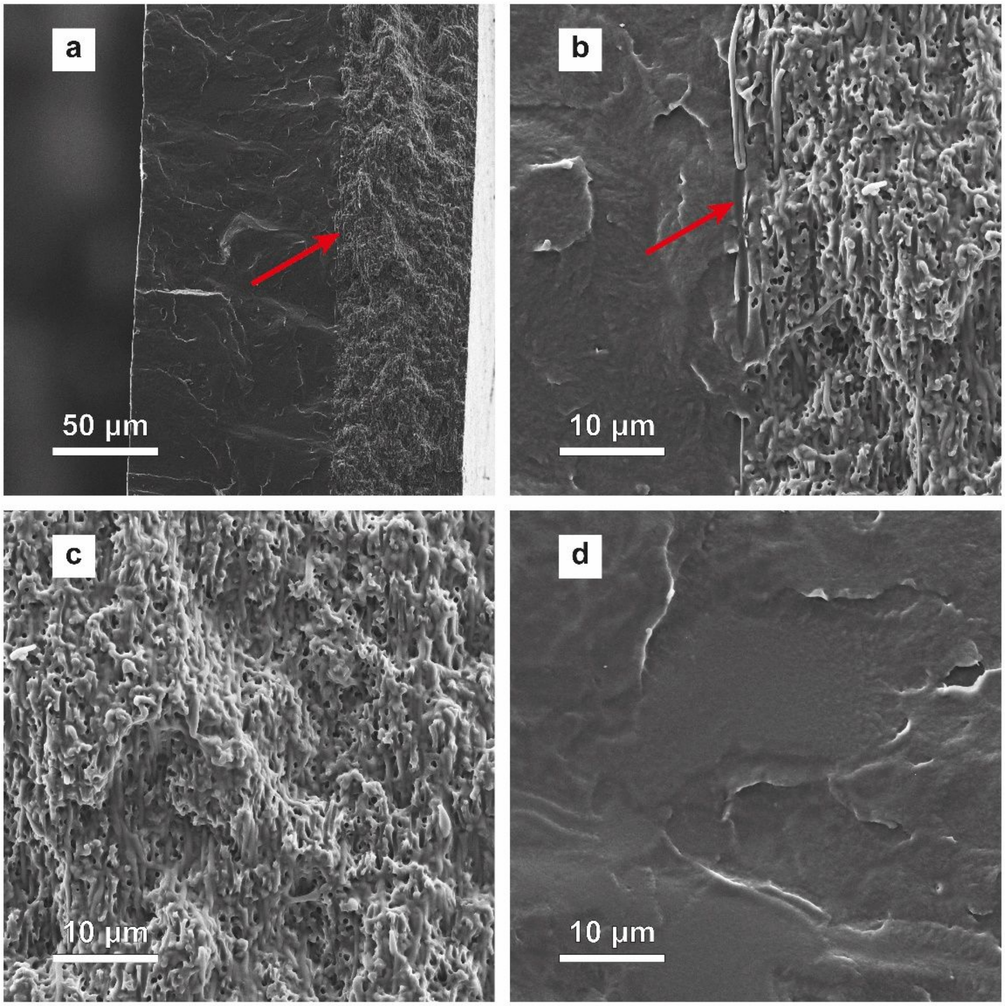

Figure S2: SEM images of the **SBS-PVA$_{20}$** composite membranes prepared with the film applier. (a-b) Cross-sections of the film (red arrows indicate the interface between the two sides of the membrane), (c) the PVA-rich side, and (d) the neat SBS layer of the composite membrane.

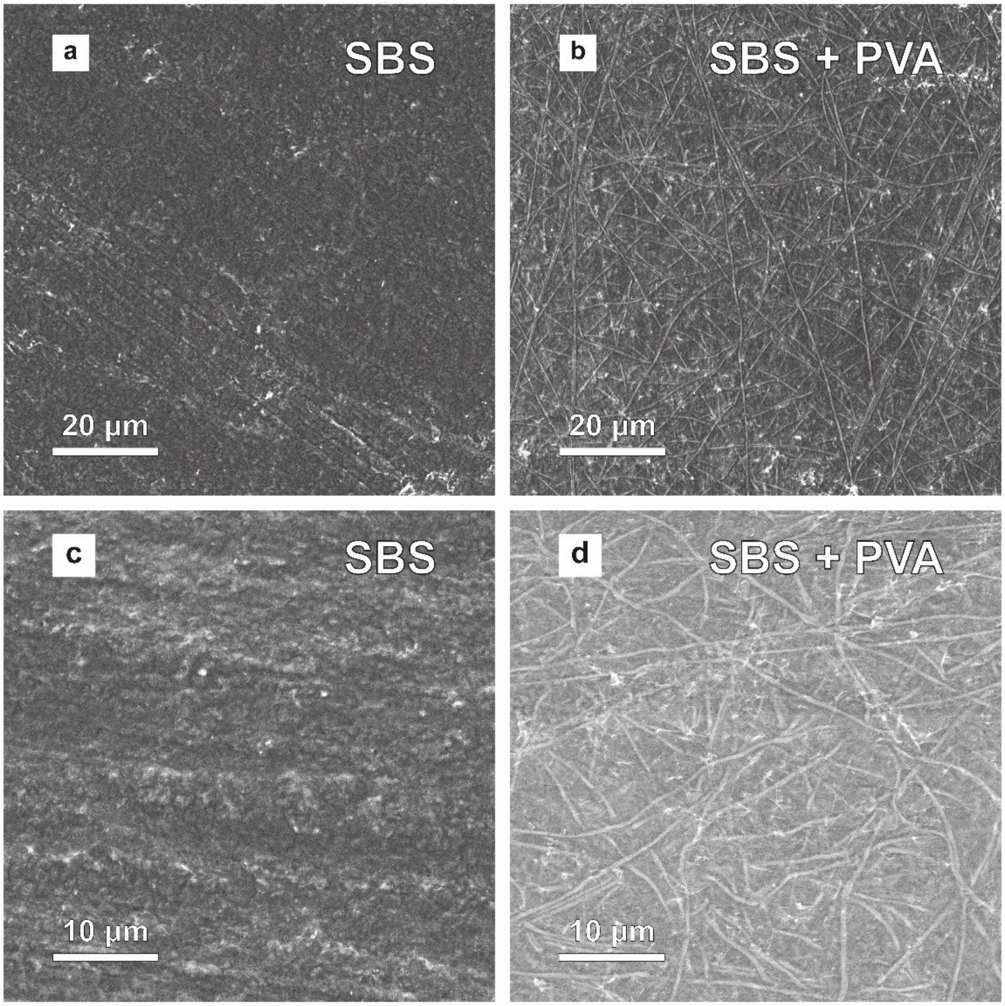

Figure S3: Top-view SEM images of the surfaces of the SBS-PVA composite membranes; (a) neat SBS side, and (b) PVA-rich side of **SBS-PVA$_{13}$** composite membrane prepared with the solution-casting method; (c) neat SBS side, and (d) PVA-rich side of **SBS-PVA$_{20}$** composite membrane prepared with the film applier.

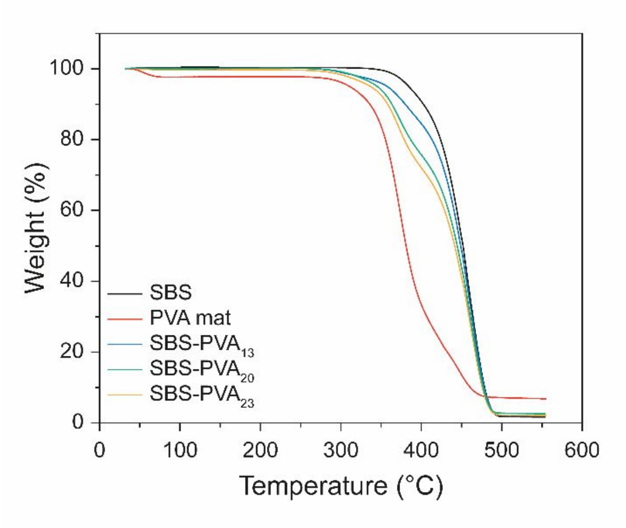

Figure S4: Thermogravimetric analysis (TGA) curves of the neat SBS, the electrospun PVA mat, and the SBS-PVA composites.

Supplementary Note 1: Calculation of the resistance to water permeation of the **SBS-PVA$_{23}$** composite membranes.

The total resistance of the composite membrane R_M is expressed by **Equation S1:**

$$R_M = \frac{l_M}{WP \times A} \qquad \text{(S1)}$$

where l_M and A are the total thickness and the transport area of the membrane. WP is the water permeability of the membrane, which assumes two different values, depending on the transport direction investigated with the wet cup method (RH_D = 100%).

The experimentally determined data are: l_M = 108 µm

A = 31.67 cm^2

and

Transport direction: SBS → PVA $WP = 1.93 \times 10^{-14}$ kg m m^{-2} s^{-1} Pa^{-1}

PVA → SBS $WP = 3.97 \times 10^{-14}$ kg m m^{-2} s^{-1} Pa^{-1}

Using **Equation S1**, we obtain:

Transport direction: SBS → PVA R_M = 1764 MPa g^{-1} s^{-1}

PVA → SBS R_M = 859 MPa g^{-1} s^{-1}

Using a series-resistance model, we can express the total resistance of the membranes R_M as the sum of the resistances of the SBS layer (R_{SBS}) and the PVA-rich side ($R_{PVA\text{-}rich}$), as shown in **Equation S2:**

$$R_M = R_{SBS} + R_{PVA-rich} \quad \textbf{(S2)}$$

Similarly, as for the total resistance R_M, we can express the resistance of the SBS layer R_{SBS} with **Equation S3:**

$$R_{SBS} = \frac{l_{SBS}}{WP_{SBS} \times A} \quad \textbf{(S3)}$$

where l_{SBS} is the thickness of the SBS layer in the composite membrane and WP_{SBS} is the water permeability of neat SBS measured with the wet cup method (RH_D = 100%).

The experimentally determined data are: l_{SBS} = 48 μm

$WP_{SBS} = 2.13 \times 10^{-14}$ kg m m^{-2} s^{-1} Pa^{-1}

Using **Equation S3** we obtain: R_{SBS} = 712 MPa g^{-1} s^{-1}

After evaluating R_M and R_{SBS}, using **Equation S2** we can derive the resistance of the PVA-rich side of the composite membrane $R_{PVA\text{-}rich}$ in the two transport directions. We obtain:

Transport direction: SBS → PVA $R_{PVA\text{-}rich}$ = 1052 MPa g^{-1} s^{-1}

PVA → SBS $R_{PVA\text{-}rich}$ = 147 MPa g^{-1} s^{-1}

These calculations were made using the average thickness value of the total membrane l_M and the SBS layer l_{SBS} in the **SBS-PVA$_{23}$** composite membrane reported in **Table 1** of the main manuscript. Similarly, for the WP of the **SBS-PVA$_{23}$** composite in the two transport directions, we assumed the average values measured with the wet cup method and reported in **Table 3** of the main manuscript. The resistances reported in **Table 3** of the main manuscript are the mean ± standard deviation of analogous calculations made using the actual values of l_M, l_{SBS} and *WP* of n = 4 different membranes.

Supplementary Note 2: Theoretical calculation of the resistance in the PVA-rich side of the **SBS-PVA$_{23}$** composite membranes.

Volume fraction of PVA in the PVA-rich side

Data: density of PVA $d_{\mathrm{PVA}} = 1.269$ g cm^{-3}

density of SBS $d_{\mathrm{SBS}} = 0.94$ g cm^{-3}

If we consider as the basis for the calculation a hypothetical membrane with a weight of 100 g and we assume a PVA weight fraction of $w_{\mathrm{PVA}} = 0.23$, we obtain:

Total PVA = 23 g → 18 cm^3

Total SBS = 77 g → 82 cm^3

Total volume = 18 + 82 = 100 cm^3

If we assume that the neat layer of SBS occupies half of the volume of the composite membrane, we obtain:

SBS layer = 50 cm^3 → 47 g

Considering the total content of SBS in the composite membrane and the SBS layer, we can calculate the SBS content in the PVA-rich side. If we assume that the PVA is present only in the PVA-rich side, we obtain:

SBS in the PVA-rich side = 77 – 47 = 30 g → 32 cm^3

PVA in the PVA-rich side = 23 g → 18 cm^3

From these values we can evaluate the volume fraction of PVA in the PVA-rich side v_{PVA} as follows:

$$v_{PVA} = \frac{18}{18 + 32} = 0.36$$

All the arrows indicated in the calculation (→) represent a conversion step between weight and volume (or vice versa) in which we used the densities reported in the specifications of the supplier (Merck).

Water permeability and resistance of the PVA-rich side

We can express the water permeability of the PVA-rich side of the composite membrane ($WP_{\mathrm{PVA\text{-}rich}}$) as the combination of the water permeability of PVA (WP_{PVA}) and SBS (WP_{SBS}) reported in **Equation S4:**

$$WP_{PVA-rich} = v_{PVA} WP_{PVA} + (1 - v_{PVA}) WP_{SBS} \quad \textbf{(S4)}$$

where v_{PVA} is the volume fraction of PVA in the PVA-rich side of the membrane evaluated before, while for the water permeability of SBS WP_{SBS} we use the value measured with the wet cup method on the reference films of neat SBS. The water permeability of PVA WP_{PVA} changes from a dry (unplasticized) to a wet (plasticized) value. If for the unplasticized value we use the water permeability measured with the dry cup method at RH_D = 60%, and for the plasticized value we use the water permeability measured with the wet cup method (RH_D = 100%), we obtain:

$$v_{\mathrm{PVA}} = 0.36$$

WP_{SBS} = 2.13× 10^{-14} kg m m^{-2} s^{-1} Pa^{-1}

Dry (unplasticized) PVA WP_{PVA} = 5.99 × 10^{-16} kg m m^{-2} s^{-1} Pa^{-1}

Wet (plasticized) PVA WP_{PVA} = 2.90 × 10^{-13} kg m m^{-2} s^{-1} Pa^{-1}

Depending on the water transport direction through the composite membrane, the PVA in the PVA-rich side is either dry (SBS → PVA) or wet (PVA → SBS). Using **Equation S4** and the corresponding value of WP_{PVA}, we obtain:

Transport direction: SBS → PVA $WP_{PVA\text{-}rich}$ = 1.38 × 10^{-14} kg m m^{-2} s^{-1} Pa^{-1}

PVA → SBS $WP_{PVA\text{-}rich}$ = 11.85 × 10^{-14} kg m m^{-2} s^{-1} Pa^{-1}

Similarly, as for the total resistance R_M, we can express the resistance of the PVA-rich side $R_{PVA\text{-}rich}$ with **Equation S5**:

$$R_{PVA-rich} = \frac{l_{PVA-rich}}{WP_{PVA-rich} \times A} \quad \textbf{(S5)}$$

where $l_{PVA\text{-}rich}$ and is the thickness of the PVA-rich side of the composite membrane and is calculated with **Equation S6**:

$$l_{PVA-rich} = l_M - l_{SBS} \quad \textbf{(S6)}$$

If for the thickness l_M and l_{SBS} we use the average values of the total thickness of the membrane and of the SBS layer in the **SBS-PVA$_{23}$** composite reported in **Table 1** of the main manuscript, we obtain: l_{PVA} = 108 μm – 48 μm = 60 μm

Using **Equation S5** and the corresponding value of $WP_{\text{PVA-rich}}$, we obtain:

Transport direction: SBS → PVA $R_{\text{PVA-rich}} = 1374$ MPa g^{-1} s^{-1}

PVA → SBS $R_{\text{PVA-rich}} = 160$ MPa g^{-1} s^{-1} .
